# Leukocyte telomere length and serum polyunsaturated fatty acids, dietary habits, cardiovascular risk factors and features of myocardial infarction in elderly patients

**DOI:** 10.1186/s12877-019-1383-9

**Published:** 2019-12-27

**Authors:** Are A. Kalstad, Sjur Tveit, Peder L. Myhre, Kristian Laake, Trine B. Opstad, Arnljot Tveit, Erik B. Schmidt, Svein Solheim, Harald Arnesen, Ingebjørg Seljeflot

**Affiliations:** 1 Center for Clinical Heart Research, Department of Cardiology, Oslo University Hospital, Ullevål, Postboks 4956 Nydalen, 0424 Oslo, Norway; 2 Faculty of Medicine, University of Oslo, Oslo, Norway; 3 Department of Cardiology, Akershus University Hospital HF, Lørenskog, Norway; 4 Department of Medical Research, Vestre Viken Hospital Trust, Bærum Hospital, Gjettum, Norway; 5 Department of Cardiology, Aalborg University Hospital, Aalborg, Denmark

**Keywords:** Telomere length, Polyunsaturated fatty acids, Diet, Elderly, Cardiovascular disease, Myocardial infarction

## Abstract

**Background:**

Telomeres are non-coding sequences at the end of eukaryote chromosomes, which in complex with associated proteins serve to protect subtelomeric DNA. Telomeres shorten with each cell division, are regarded as a biomarker for aging and have also been suggested to play a role in atherosclerosis and cardiovascular disease (CVD). The aim of the present study was to explore the associations between leukocyte telomere length and serum polyunsaturated fatty acids, diet, cardiovascular risk factors and features of myocardial infarction (MI) in elderly patients.

**Methods:**

The material is based upon the first 299 included patients in the OMEMI trial, where patients aged 70–82 years of age are randomized to receive omega-3 supplements or corn oil (placebo) after MI. Patients were included 2–8 weeks after the index MI. DNA was extracted from whole blood, and leukocyte telomere length (LTL) was analyzed by qPCR and reported as a number relative to a reference gene. Serum long chain polyunsaturated fatty acid (LCPUFA) content was analyzed by gas chromatography. Diet was evaluated with the validated SmartDiet food frequency questionnaire. Medical records, patient interviews and clinical examination provided previous medical history and anthropometric data. Non-parametric statistical tests were used.

**Results:**

Median (25, 75 percentile) LTL was 0.55 (0.42, 0.72). Patients had a median age of 75 years, 70.2% were male and 45.2% used omega-3 supplements. There was a weak, but significant correlation between LTL and linoleic acid (*r* = 0.139, *p* = 0.017), but not with other LCPUFAs. There was a trend towards longer telomeres with a healthier diet, but this did not reach statistical significance (*p* = 0.073). No associations were found between LTL and CVD risk factors or features of MI.

**Conclusions:**

In our population of elderly with a recent myocardial infarction LTL was associated with linoleic acid concentrations, but not with other LCPUFAs. Patients with a healthy diet tended to have longer telomeres. The limited associations may be due to age and the narrow age-span in our population. Further studies, designed to detect longitudinal changes should be performed to explore the role of telomeres in cardiovascular aging.

**Trial registration:**

Clinical trials no. NCT01841944, registration date April 29, 2013.

## Background

Age is the most important risk factor for cardiovascular disease (CVD) and the prevalence of age-related diseases is increasing with the general ageing of the population [1]. Several molecular pathways of ageing related to CVD have been suggested, including oxidative stress, inflammation and remodeling [2–4]. Lately, there has been great interest in the importance of telomeres and their shortening [5, 6] both as related to the ageing process and their role in CVD.

Telomeres are repeating hexameric segments (TTAGGG) of DNA and associated proteins, and form the ends of chromosomes of eukaryotic cells. They play an important role in stabilizing the chromosome and protect the ends against degradation or end-to-end fusion during DNA damage and repair. As DNA polymerase is unable to replicate the ends of chromosomes, their length is shortened with each cell division. The telomere loss over time is age-dependent in humans [7], ranging 30–200 base pairs per cell cycle [8]. After reaching a certain length, the cells cease to divide, leading to cellular senescence [9]. Telomere shortening is counteracted by the enzyme telomerase, which has the capability to elongate telomeres [10]. In addition, telomere shortening has been shown to be influenced by modifiable factors such as lifestyle and environmental risk factors [11, 12]. Leukocyte telomere length (LTL) and their attrition rate have been associated with different markers of atherosclerosis in some studies [6, 13–15], while others have demonstrated more ambiguous results [16, 17]. Additionally, a relationship between telomere length and myocardial infarction (MI) has been reported [18, 19].

Long chain polyunsaturated fatty acids (LCPUFA) have been proposed to influence LTL, possibly through effects on oxidative stress and telomerase activity [20]. An observational study by Farzaneh-Far R et al. [21] demonstrated a significant reduced shortening with higher baseline levels of eicoapentaenoic acid (EPA) and docosahexaenoic acid (DHA) over a period of 6 years in a CVD population [21]. Reduced shortening of telomeres was also reported with decreasing n-6/n-3 ratio in a supplementation study in a healthy middle-aged population [22].

The aim of the present study was to further explore the relationship between LTL and selected commonly studied LCPUFAs and diet in elderly survivors of myocardial infarction (MI). In addition, any associations of LTL with risk cardiovascular risk factors, MI characteristics and markers of myocardial injury and dysfunction were explored.

## Material and methods

### Study design

This paper presents a cross-sectional substudy of data obtained at time of inclusion in the ongoing OMega-3 fatty acids in Elderly patients with Myocardial Infarction (OMEMI) trial [23], in which elderly survivors of AMI are randomized to 2 years intervention with either 1.8 g marine n-3 fatty acids in the form of Pikasol capsules (Axellus AS, Oslo/Orkla Health, Norway) or corn oil placebo. The present investigation was performed in the first 299 consecutively enrolled patients.

The project was performed in accordance with the Declaration of Helsinki, approved by the Regional Ethical Committee South East with (Reference no.: 2012/1422), and registered at ClinicalTrials.gov (NCT01841944). All patients gave written informed consent to participate.

### Patient population

Patients were recruited at three hospitals in the Oslo region of Norway; the Oslo University Hospital, Ullevål, Oslo, Vestre Viken Hospital Trust, Bærum and Akershus University Hospital, Lørenskog. Patients were screened during their initial hospital admission. Inclusion criteria were patients who had undergone MI, age between 70 and 82 years, and ability to understand verbal and written patient information in Norwegian language and provide written consent. Exclusion criteria were documented intolerance for n-3 fatty acids, and additional disease states thought to influence survival and/or ability to adhere to study medication and follow up for the study period. Previous regular intake of n-3 supplements was not an exclusion criterion, as such intake is fairly common in the general Norwegian population, and exclusion would introduce a selection bias to the study. Patients were included two to 8 weeks after hospital discharge. The inclusion period for the 299 patients was November 2012 to November 2014. The study design has previously been described in detail [23].

Previous medical history and data regarding the index MI were retrieved from hospital records, including the classification of the MI as ST-segment elevation MI (STEMI) or non-ST-segment MI (NSTEMI), and peak Troponin T levels occurring from sequential measures during hospital stay. Echocardiography was performed as part of the clinical management in a total of 162 patients, and left ventricular ejection fraction (LVEF) was registered. Previous hypertension was defined as previous diagnosis of hypertension, and previous hyperlipidemia was defined as previous diagnosis or treatment with lipid lowering drugs. Current smoking was defined as current smoking or cessation no more than 3 months prior to inclusion.

### Diet registration

The patients’ diet was assessed using the SmartDiet form which is a validated food frequency questionnaire, previously described [24]. The SmartDiet form was filled out once, and is based on the patients’ recollection of intake of different food groups, with a special emphasis on fats. Each of the items are scored, and summarized to a total score ranging from 15 to 45 points. The scores are further divided into three groups, where a score of 15–27 is considered an unhealthy diet (designated poor), a score of 28–35 a diet should be improved (designated intermediate), and a score of 36–45 points is considered a healthy diet (designated healthy). As the subdivision into the three arbitrary categories may limit the discovery of association, data from SmartDiet score were also explored as a continuous variable in the present study.

### Laboratory analysis

Venous blood samples were drawn in fasting state between 08:00 and 11:00 at inclusion and routine laboratory analyses were performed by conventional methods.

Serum was prepared within 1 h by centrifugation at 2000 x g for 10 min and kept frozen at − 80 °C until analysis of the fatty acid profile. EDTA whole blood was drawn and kept frozen at − 80 °C until DNA extraction for LTL analysis.

Fatty acids composition in serum phospholipids was analyzed by gas chromatography and expressed as % weight of total fatty acids, as previously described [25]. Briefly, serum lipids were extracted according to the Folch procedure [26]. The organic phase containing total lipids was collected and another extraction was performed. Separation of the phospholipid fatty acid fraction from total lipids was performed as described by Burdge et al. [27]. Methylation of the fatty acids was performed before being analyzed by gas chromatography using a Varian 3900 gas chromatograph equipped with a CP-8400 autosampler, a flame ionization detector and a high-polarity polyester CP-Sil 88 60 m × 0.25 mm capillary column (Varian, Middleburg, The Netherlands).

The content expressed as % weight of serum phospholipids of the LCPUFAs linoleic acid (LA) 18:2 n-6, arachidonic acid (AA) 20:3 n-3, alpha-linolenic acid (ALA) 18:3 n-3, eicosapentaenoic acid (EPA) 20:5 n-3 and docosahexaenoic acid (DHA) 22:6 n-3 and the ratio of the n-6 to n-3 LCPUFAs were used for the present study, as well as the ratio of the selected n-6 PUFAs to the selected n-3 PUFAs. These have all previously been extensively studied in relation to CVD [25].

### Analysis of LTL

Leukocytes are a commonly used source for analysis of telomere length in clinical studies, as they are easily obtained from the circulation, also used in our study. These have been shown to have acceptable correlation to telomere lengths in other tissues.

DNA was extracted from EDTA whole blood by the same lot of the QIAamp DNA Blood Mini Kit throughout the study (Qiagen Gmbh, Hilden, Ger) and according to the manufacturers instruction. DNA purity and quantity was tested on the NanoDrop, ND-1000 (Saveen Werner, Sweden) and samples were stored at − 80 °C until LTL analyses.

All DNA samples were diluted to a final concentration of 2 ng/μL and LTL was measured by singleplex quantitative real-time PCR [28]. PCR amplification was performed on the VIIa7 PCR Instrument with the QuantStudio Real-Time Software (Applied Biosystems by Life Technologies Foster City, CA, USA),using telomere-specific primers (Invitrogen by Thermo Fisher Scientific, Waltham, MA, US) (Additional file 1: Table S1) and GoTaq®qPCR Master Mix (Promega, Madison, WI, US). LTL was relatively quantified (RQ) to the single-copy-gene (SCG) 36B34 (Invitrogen by Thermo Fisher Scientific) (Additional file 1: Table S1) and to an internal reference sample. The primers for both targets were diluted to a final concentration of 4 pmol /μl, and PCR conditions were as follow; an initial step at 95 °C for 10 min followed by 40 cycles of 95 °C for 15 sek and 60 °C for 1 min. All samples were run in triplicates [28].. Amplification efficacy for both targets were equal, and approximately 100%. Individual amplification curves for all samples of both assays (LTLs and SCG) were carefully validated. Technical replicates with a standard deviation (SD) exceeding 0.5 Ct or outliers, if diversely to the other replicates were excluded from the analysis. A template negative control was included in each run. The intra- and inter assay coefficient of variation (CV) for the LTL Ct values were; 0.37 and 2.1%, and for the SCG Ct values 0.20 and 0.40% (based on mean CV value between replicates on 1 plate and multiple plates, respectively). The inter-run CV for the T/S ratio (LTL Ct signal divided on the CT mean of the reference sample) was 2.09% (based on mean CV for triplicates run on different plates). The inter-run CV for the exponentially calculated T/S ratio was 13.2%, and when relatively calculated to the reference sample, the CV was 13.3% (Additional file 3: Methods).

### Statistical analysis

Continuous variables are presented as mean ± SD or median (25th, 75th percentiles) as appropriate. Categorical data are presented as numbers and/or percentages. As most data were non-normally distributed non-parametric statistical tests were performed. Differences between groups were examined by Kruskal-Wallis or Mann-Whitney U test. For correlation analyses Spearman’s rho were used.

A *p*-value < 0.05 (two-tailed) was considered statistically significant. All statistical analyses were performed using SPSS ver. 24, IBM Corporations.

## Results

Baseline characteristics of the patient cohort (*n* = 299) are presented in Table 1.The median age of was 75 (72, 78) years. Males comprised 70.2% of the population. All patients were of Caucasian ethnicity. Other cardiovascular risk factors were prevalent, with 60.9% diagnosed with hypertension or used anti-hypertensive medications, 47.8% diagnosed with hyperlipidemia or on lipid-lowering agents and 23.1% were diagnosed with diabetes mellitus. Pre-existing coronary artery disease was reported in 45.2% of patients prior to the index MI. A total of 40 patients (13.4%) had diagnosis of heart failure, either preexisting or diagnosed during or after the index hospitalization. LVEF < 50% was recorded in 52 patients (32.1% of 162). NSTEMIs constituted 68.6% of cases and STEMIs the remaining 31.4%.

**Table 1:** Characteristics of the study cohort. Data are presented as number (%) or median values (25, 75 percentiles)

Age (years)(range)	75 (70,82)
Males	210 (70.2)
BMI (kg/m^2^)	25.6 (23.8, 28.3)
Systolic BP (mmHg)	140 (125, 151)
Diastolic BP (mmHg)	74 (67, 80)
Current smokers	41 (13.7)
Previous hyperlipidemia	156 (47.8)
Previous hypertension	182 (60.9)
Diabetes mellitus	69 (23.1)
Previous chronic kidney disease^1^	15 (5.1)
Previous heart failure	16 (5.4)
Previous coronary artery disease	135 (45.2)
Previous ischaemic stroke	21 (7.0)
NSTEMI/STEMI	68.6 / 31.4 (205 / 94)
3-vessel disease^2^	61 (21.3)
Maximum Troponin T (ng/L)	700 (153, 2500)
NT-proBNP (ng/L)	634 (279, 1374)
LVEF < 50%^3^	52 (32.1)
Taking n-3 FA supplement	135 (45.2)

*BMI* Body Mass Index; *NSTEMI* Non-ST-segment elevation myocardial infarction; *STEMI* ST-segment elevation myocardial infarction; *NT-proBNP* N-terminal pro-Brain natriuretic peptide; *LVEF* Left ventricle ejection fraction; *FA* fatty acids

^1^creatinine > 150 μmol/L

^2^ of *n* = 286 with angiography

^3^ of *n* = 162 with echocardiography

### Leukocyte telomere length (LTL)

LTL in the total cohort ranged from 0.11 to 1.55 and was skewed towards shorter telomeres, with a median of 0.55 (0.42, 0.72).

### LTL and fatty acids

Levels of the selected LCPUFAs presented as % weight of total serum phospholipids and the n-3/n-6 ratio in the total population and stratified by self-reported use of n-3 polyunsaturated fatty acid (PUFA) supplementation are presented in Table 2. Patients who reported regular intake of n-3 PUFA supplements (*n* = 135), had significantly higher levels of both EPA and DHA than those who did not, and also lower levels of AA (all *p* < 0.001). The n-6/n-3 ratio was also lower in patients taking n-3 PUFA supplements (*p* < 0.001).

**Table 2:** Levels of selected PUFAs as % weight of total serum phospholipids, in total and according to reported intake of n-3 supplements

	Total	n-3 supplementation		p
		Yes	No	
Linoleic acid (LA) 18:2 n-6	19.00 ± 3.13	18.83 ± 3.02	19.16 ± 3.22	0.401
Arachidonic acid (AA) 20:3 n-3	9.65 ± 2.22	8.96 ± 1.89	10.24 ± 2.32	< 0.001
Alpha-linolenic acid (ALA) 18:3 n-3	0.23 ± .08	0.23 ± .08	0.23 ± .09	0.242
Eicosapentaenoic acid (EPA) 20:5 n-3	2.74 ± 1.38	3.20 ± 1.54	2.34 ± 1.10	< 0.001
Docosahexaenoic acid (DHA)	5.71 ± 1.43	6.31 ± 1.38	5.21 ± 1.27	< 0.001
n-6/n-3 ratio	3.64 ± 1.29	3.10 ± 1.04	4.09 ± 1.31	< 0.001

Mean ± SD are given. *p*-values are given for difference between n-3 supplementation or not

**Fig. 1:**
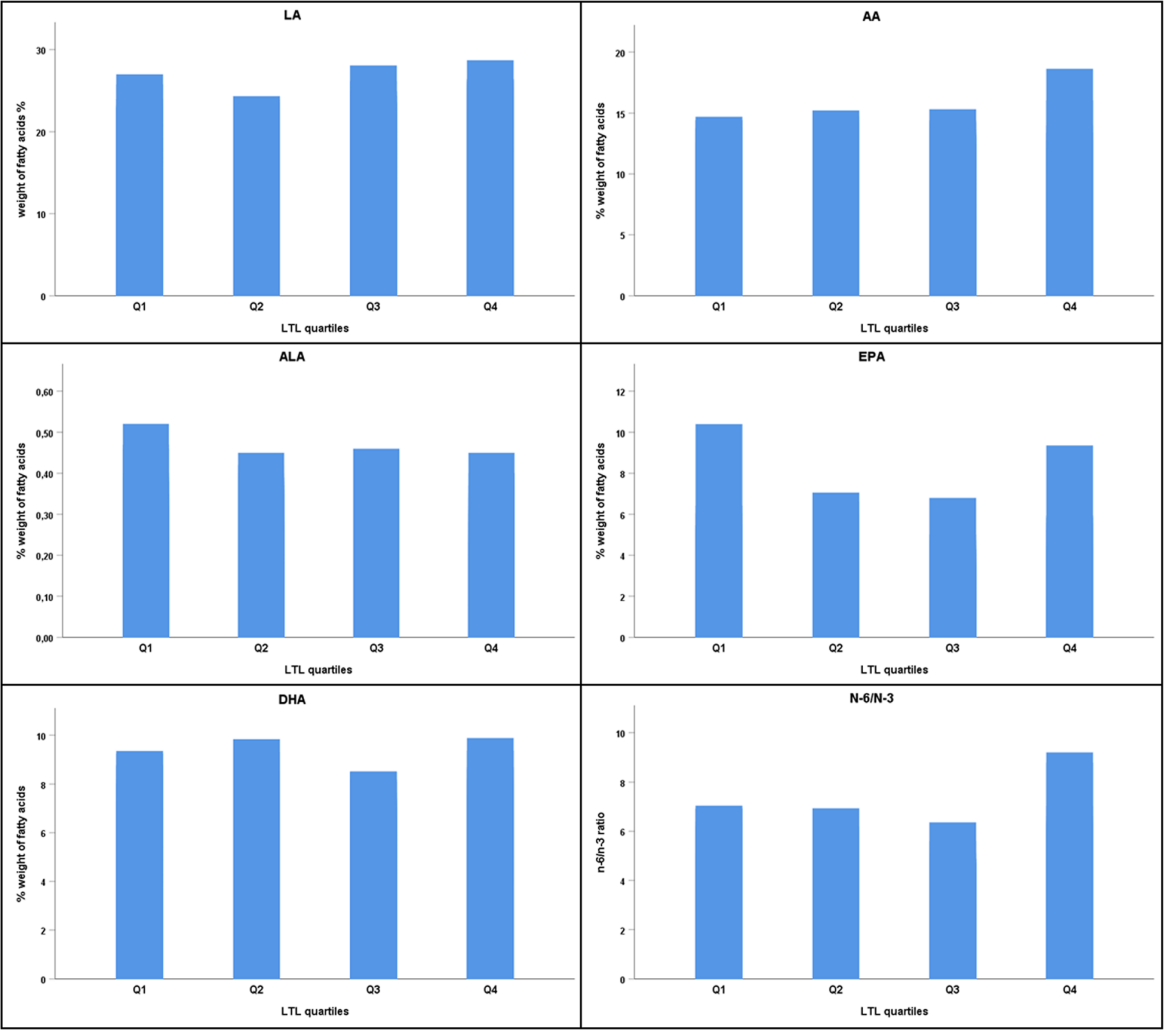
Levels of the selected fatty acids (weight %) and the n-6/n-3 ratio across quartiles of LTL. LA: Linoleic acid, ALA: Alpha-linolenic acid, EPA: Eicosapentaenoic acid, AA: Arachidonic acid DHA: Docosahexaenoic acid. All p > 0.1 (Kruskal-Wallis test for differences between quartiles)

Levels of LTL correlated weakly, but statistically significantly to the content of LA in serum phospholipids (*r* = 0.139, *p* = 0.017). Neither of the remaining investigated LCPUFAs, nor the n-6/n-3 ratio correlated significantly to LTL (r ranging from − 0.107 to 0.087 and p ranging from 0.067 to 0.958) (Table 3). Associations were also examined for differences in fatty acids levels across quartiles of LTL, with no significant differences (*p* > 0.1) (Fig. 1). When excluding patients using n-3 supplementation, the correlation between LTL and LA was borderline significant (*r* = 0.141, *p* = 0.074) (Additional file 2: Table S2), whereas the differences across quartiles of LTL did not change (all *p* > 0.1).

**Table 3:** Coefficients of correlations (r) between LTL and serum phospholipid fatty acid levels

Fatty acid	Spearman’s rho	p
Linoleic acid (LA) 18:2 n-6	0.139	0.017
Arachidonic acid (AA) 20:3 n-3	0.014	0.812
Alpha-linolenic acid (ALA) 18:3 n-3	−0.107	0.067
Eicosapentaenoic acid (EPA) 20:5 n-3	−0.080	0.167
Docosahexaenoic acid (DHA) 22:6 n-3	0.003	0.958
n-6/n-3 ratio	0.087	0.137

### LTL and diet score

**Fig. 2:**
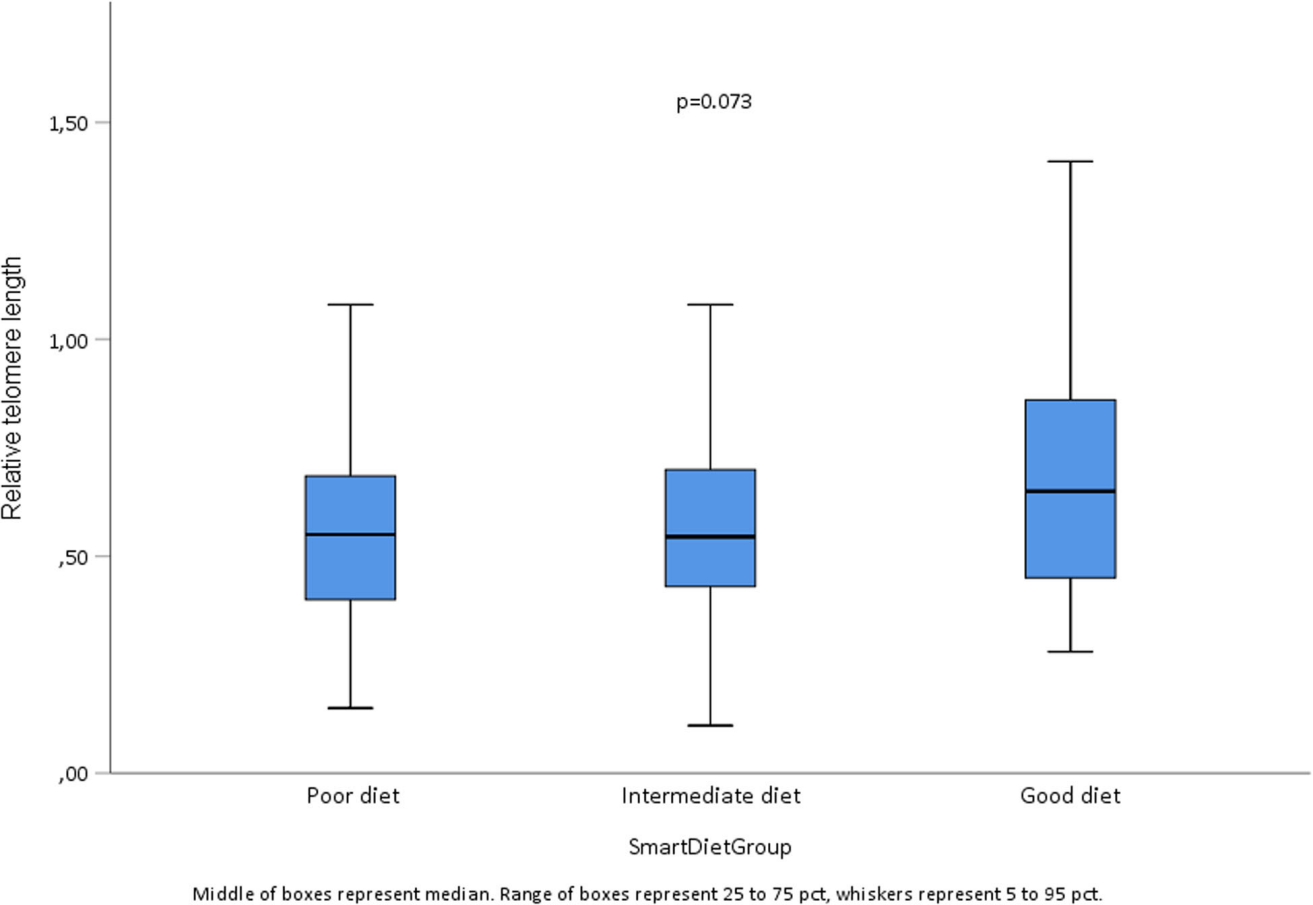
Median LTL levels across SmartDiet score groups. Poor diet (*n* = 71) Score ≤ 27, intermediate diet (*n* = 142) Score 38–35 and healthy diet (*n* = 18) Score ≥ 36

The mean SmartDiet score was 29.4 (range 15, 40), with 71 (30.7%) in Group 1 (poor), 142 (61.5%) in Group 2 (intermediate) and 18 (7.8%) in Group 3 (healthy). The respective median (25, 75%) levels of LTL were 0.55 (0.39, 0.69), 0.55 (0.43, 0.70) and 0.65 (0.45, 0.87) (Fig. 2). Despite a trend for longer LTLs in the healthy group, the difference did not reach statistical significance (*p* = 0.073). No statistically significant correlation between LTL and score obtained by SmartDiet as a continuous variable was observed (*r* = 0.043, *p* = 0.52).

### LTL and traditional cardiovascular risk factors

LTL was not significantly correlated with age, neither for the patient group as a whole, nor when divided by gender (all p > 0.1). There were also no significant difference between males and females, 0.55 (0.43, 0.74) vs 0.54 (0.38, 0.67) (*p* = 0.30).

To explore any association between LTLs and traditional risk factors, levels of LTL were dichotomized according to values below or above median level (0.55). As show in Table 4, no significant differences were observed according to values below or above median level in number of patients with the risk factor or not.

**Table 4:** Cardiovascular risk factors a) and features of myocardial infarction b) according to dichotomized LTL levels below or above the median (0.55)

a)	LTL < 0.55	LTL ≥0.55	p
Male	100 (47.8%)	109 (52.2%)	0.486
Previous hypertension^a^	91 (50.3%)	90 (49.7%)	0.630
Previously hyperlipidemia^b^	68 (47.9%)	74 (52.1%)	0.675
Current smoker	23 (56.1%)	18 (43.9%)	0.338
Diabetes mellitus	31 (44.9%)	38 (55.1%)	0.422
Chronic kidney disease^c^	9 (60.0%)	6 (40.0%)	0.381
Previous coronary artery disease	63 (47.0%)	71 (53.0%)	0.503
BMI (kg/m^2^)	25.8 (23.6, 28.7)	25.5 (24.0, 28.0)	0.353
b)			
STEMI	41 (43.6%)	53 (56.4%)	0.194
Peak Troponin T (ng/l)	814 (146, 2355)	942 (170, 3013)	0.346
NT-proBNP (ng/l)	617 (245, 1281)	689 (321, 1431)	0.381
LVEF< 50%	54 (49.1%)	56 (50.9%)	0.637

*BMI* Body Mass Index; *STEMI* ST-segment elevation myocardial infarction; *NT-proBNP* N-terminal pro-Brain natriuretic peptide; *LVEF* Left ventricle ejection fraction

*P*-values refer to difference between groups with LTL below vs above median level; Pearson’s chi square or Mann-Whitney U test

^a^ Defined as previous diagnosis of hypertension

^b^Defined as previous diagnosis of hyperlipidemia

^c^Creatinine > 150 μmol/L

### LTL and features of myocardial infarction

Patients with STEMI had numerically longer LTL compared to NSTEMI (0.57 (0.43, 0.75) vs 0.53 (0.42, 0.72), but not statistically significant (*p* = 0.89). LTLs dichotomized by median level showed no significant difference between values below or above the median in any marker of myocardial injury or myocardial function, as shown in Table 4. There was also no difference in LTL of patients presenting with heart failure at any time.

## Discussion

The findings in this study of telomere lengths in elderly patients with a recent MI were predominantly neutral. A weak, but significant correlation between serum levels of linoleic acid and LTL, and a borderline relationship between LTL and dietary habits were found, whereas no significant relation to conventional cardiovascular risk factors or features of MI could be demonstrated.

Previous studies investigating the associations between LCPUFAs and LTL have shown that these fatty acids affect telomere attrition rate. However, in accordance with our findings no associations on a cross-sectional level have been demonstrated. Farzaneh-Far et al. found that the higher quartiles of serum EPA+ DHA were associated with reduced telomere shortening over 6 years in patients with coronary heart disease [21]. A decreasing n-6/n-3 ratio was associated with reduced shortening in a 4 months intervention study on healthy, sedentary overweight middle-aged and older individuals [22]. Associations between EPA and DHA and LTL have been attributed mainly to reduced oxidative stress [21, 22], but also a possible effect on telomerase activity [20].

The positive correlation between LA and LTL levels found in our study is, although significant, very weak. In contrast to this finding, an inverse association between LTL levels and LA was demonstrated, however, in a younger female population and with LA data derived from a dietary questionnaire [29]. It might, nevertheless, be speculated that a mechanistic influence of PUFAs on telomere attrition may differ across age and gender.

Although not statistically significant, a healthier diet appeared to be associated with longer telomeres in our study. Due to the low number of patients in the healthy diet group, the analysis may have been underpowered to detect a significant difference. In line with our results, dietary fiber intake, associated with a healthy diet, has been reported to be positively associated with LTL [29]. There is also some evidence to support that adherence to a Mediterranean diet is associated with longer telomeres [12, 30, 31]. The SmartDiet score is primarily used to explore the qualitative composition of the diet with a focus on lipids, and cannot directly assess total caloric intake. This may be important when discussing possible mechanisms and impact on telomere length, such as oxidative stress.

LTL length has been associated with atherosclerotic disease states in several studies [32–34], however, associations with various risk factors are less consistent [35–37]. The most surprising finding in our study was the lack of an association between age and LTL length as this is firmly established in a multitude of other studies. This might be due to the narrow age span of the population, but also to the fact that the population per se was older. While the shortening of telomeres with age is well-established, an attenuated telomere shortening with older age has also been discussed [38, 39]. Another surprising finding was the lack of gender difference, which has been shown in several studies [38, 39]. Although men and women seem to have equal telomere lengths at birth, male telomeres have been reported to shorten faster [40]*.* A general problem with studies on telomeres in humans is that telomeres have a very large inter-individual variation [41], and that the long life span of humans has not yet permitted studies of the full lifespan dynamics of telomeres from birth to old age.

We could not find any associations between LTL and traditional cardiovascular risk factors. Such associations have been demonstrated in some studies [35, 37, 42], but there is no consistent pattern, and other studies have also shown neutral results [17, 36]. Our population having a recent MI, were heavily medicated at the time of blood sampling, however, we do not have data on previous medication, which may have influenced the results. Nevertheless, the lack of association between LTL and established risk factors may be interpreted as supporting LTL length per se as a risk factor for CVD and not acting through other factors.

We could also not show any relation between LTL and the degree of myocardial injury or function. There are limited previous data in this regard. Studies have associated shorter LTL with chronic heart failure [43, 44], and one study has shown poorer prognosis in chronic heart failure with shorter LTL [45]. To our knowledge, no report regarding LTL and heart failure in the setting of MI in humans has been published, and also no data on LTL and degree of myocardial necrosis.

### Limitations

A significant limitation of the present study is the lack of a control population at the same age, but free from known CVD. Also, as mentioned, any medication used before the index infarction, might have influenced the results. Related to marine n-3 fatty acids, measure of telomerase activity might have added to the results. Measures of myocardial injury and function were limited to peak troponin T, NT-proBNP and LVEF by routine echocardiography. A significant strength with regard to the influence of LCPUFAs, however, is the individual measurement of serum fatty acids.

## Conclusion

In our study on older patients with a recent MI, no significant associations between LTL and marine LCPUFAs were found. However, a weak correlation to linoleic acid was noted. Patients with a healthy diet tended to have longer telomeres, indicating the importance of life-style factors. The lack of associations between LTL and traditional cardiovascular risk factors and features of myocardial infarction might be due to the older, age-homogeneous population. Further, especially longitudinal studies are warranted.

## Supplementary information

**Additional file 1: Table S1.** Primer sequences for the telomere and single copy gene analyses.

**Additional file 2: Table S2.** Coefficients of correlations (r) between LTL and serum phospholipid fatty acid levels, patients with previous regular intake of n-3 supplements excluded from analysis.

**Additional file 3.** Methods. Description regarding CV calculations.

## Data Availability

The datasets used during the current study are available from the corresponding author on reasonable request.

## Abbreviations

AA: Arachidonic acid
ALA: Alpha-linolenic acid
BMI: Body Mass Index
CV: Coefficient of variation
CVD: Cardiovascular disease
DHA: Docosahexaenoic acid
EPA: Eicoapentaenoic acid
FA: Fatty acids
LA: Linoleic acid
LCPUFA: Long chain polyunsaturated fatty acid
LTL: Leukocyte telomere length
LVEF: Left ventricular ejection fraction
MI: Myocardial infarction
NSTEM: Non-ST-segment myocardial infarction
NT-proBNP: N-terminal pro-Brain natriuretic peptide
OMEMI: OMega-3 fatty acids in Elderly patients with Myocardial Infarction
PUFA: Polyunsaturated fatty acid
qPCR: Quantitative polymerase chain reaction
RQ: Relative quantification
SCG: Single-copy-gene
SD: Standard deviation
STEMI: ST-segment elevation myocardial infarction
