# Supplementary material for: Leukocyte telomere length and serum polyunsaturated fatty acids, dietary habits, cardiovascular risk factors and features of myocardial infarction in elderly patients
Source: BMC Geriatr. 2019 Dec 27;19:376. doi: 10.1186/s12877-019-1383-9 (PMC6935134; doi:10.1186/s12877-019-1383-9)
Supplement: Supplementary file 1 — Additional file 1: Table S1. Primer sequences for the telomere and single copy gene analyses. [file 12877_2019_1383_MOESM1_ESM.docx]

Supplementary Table 1 Primer sequences for the telomere and single copy gene analyses.

| PCR primers | Oligomer squences (5’ to 3’) |
| --- | --- |
| Telomere fw. | CAG CAA GTG GGA AGG TGT AAT CC |
| Telomere rev. | GGC TTG CCT TAC CCT TAC CCT TAC CCT TAC CCT TAC CCT |
| Single copy gene (36B4) fw | CAG CAA GTG GGA AGG TGT AAT CC |
| Single copy gene (36B4) rev | CCC ATT CTA TCA TCA ACG GGT ACA A |

fw; forward, rev; reverse
