# Supplementary material for: Leukocyte telomere length and serum polyunsaturated fatty acids, dietary habits, cardiovascular risk factors and features of myocardial infarction in elderly patients
Source: BMC Geriatr. 2019 Dec 27;19:376. doi: 10.1186/s12877-019-1383-9 (PMC6935134; doi:10.1186/s12877-019-1383-9)
Supplement: Supplementary file 2 — Additional file 2: Table S2. Coefficients of correlations (r) between LTL and serum phospholipid fatty acid levels, patients with previous regular intake of n-3 supplements excluded from analysis. [file 12877_2019_1383_MOESM2_ESM.docx]

Supplementary Table 2 Coefficients of correlations (r) between LTL and serum phospholipid fatty acid levels, patients with previous regular intake of n-3 supplements excluded from analysis.

| Fatty acid | Spearman’s rho | p |
| --- | --- | --- |
| Linoleic acid (LA) 18:2 n-6 | 0.141 | 0.074 |
| Arachidonic acid (AA) 20:3 n-3 | -0.150 | 0.058 |
| Alpha-linolenic acid (ALA) 18:3 n-3 | 0.045 | 0.576 |
| Eicosapentaenoic acid (EPA) 20:5 n-3 | -0.105 | 0.186 |
| Docosahexaenoic acid (DHA) 22:6 n-3 | 0.016 | 0.839 |
| n-6/n-3 ratio | 0.124 | 0.119 |
