# Supplementary material for: Leukocyte telomere length and serum polyunsaturated fatty acids, dietary habits, cardiovascular risk factors and features of myocardial infarction in elderly patients
Source: BMC Geriatr. 2019 Dec 27;19:376. doi: 10.1186/s12877-019-1383-9 (PMC6935134; doi:10.1186/s12877-019-1383-9)
Supplement: Supplementary file 3 — Additional file 3. Methods. Description regarding CV calculations. [file 12877_2019_1383_MOESM3_ESM.docx]

Supplementary methods

The calculated intra- and inter-run CV for telomeres (T) and the SCG (S) are based on mean CV values between replicates on one plate and multiple plates, respectively.

When calculating the T/S ratio taking into account the exponential amount of the PCR product with increasing cycle number, the equation:

*[2^Ct(telomeres)/2^Ct(SCG)]^-1=2^-deltaCt*

has been performed, giving us an inter-assay CV for the T/S ratio of 13.2% (based on triplicates run on different plates).

When a reference sample is included in the equation, the exponential telomere and SCG values were calculated separately from the equation:

*2^(Ct reference - Ct sample)*

and the T/S ratio were calculated based on the obtained T and S values, giving us an inter-assay CV for the T/S ratio of 13,31%.
